# Protective effects of naringenin and naringin against bisphenol A–induced male reproductive toxicity: mechanistic and preclinical evidence

**DOI:** 10.1186/s12610-026-00330-4

**Published:** 2026-10-13

**Authors:** Okon Michael Ben, Ugwu Okechukwu Paul-Chima, Idehen Charles Iyore, Olorunnisola Sinbad Olubukola, Mounmbegna Philippe, Aja Patrick Maduabuchi

**Affiliations:** 1 Department of Biochemistry, Faculty of Biomedical Sciences, Kampala International University-Western Campus, Ishaka, Uganda; 2 Department of Research, Publication and Extension, Kampala International University, Ishaka-Bushenyi, Uganda; 3 School of Allied Health Sciences, Kampala International University-Western Campus, Ishaka, Ishaka-Bushenyi Uganda; 4 Centre for Artificial Intelligence and Multidisciplinary Innovations Studies, Department of Auditing, College of Accounting Science, University of South Africa, Pretoria, South Africa

**Keywords:** Bisphenol A, Naringenin, Naringin, Male fertility, Sperm quality, Endocrine disruption, Oxidative stress, Mitochondrial dysfunction, Offspring reproductive programming, Bisphénol A, Naringénine, Naringine, Fertilité masculine, Qualité spermatique, Perturbation endocrinienne, Stress oxydatif, Dysfonction mitochondriale, Programmation reproductive de la descendance

## Abstract

Bisphenol A (BPA) has been associated with altered male reproductive hormones and semen endpoints in epidemiological studies and produces endocrine, oxidative, mitochondrial, apoptotic, and developmental injury in experimental models. This narrative review evaluates BPA-specific evidence for naringenin and naringin separately from supportive evidence in other reproductive stress models. It also distinguishes directly exposed F1 outcomes from transgenerational inheritance and links sperm endpoints to hormones, histology, and molecular measures. This narrative review synthesizes evidence published mainly between January 2019 through September 2026, while retaining older studies of direct mechanistic relevance. Evidence was identified through targeted searches of PubMed, Scopus-indexed sources, Google Scholar, and publisher databases and was organized by exposure timing, biological mechanism, model type, and reproductive endpoint. The strongest evidence for BPA-related male reproductive toxicity comes from convergent cellular and animal data supported by heterogeneous human observational studies. The strongest evidence for naringenin or naringin is preclinical and includes BPA-specific Sertoli-cell and rat models as well as related oxidative, metabolic, and phthalate stress models. Across the broader preclinical literature, the flavanones modify redox, mitochondrial, inflammatory, and steroidogenic endpoints, but differences in model, dose, route, and bioavailability prevent quantitative comparison. The evidence supports mechanistic research, not clinical supplementation, and exposure reduction remains the primary public-health approach.

## Highlights

BPA affects male reproduction through endocrine, redox, mitochondrial, apoptotic and developmental pathways.

Naringenin and naringin evidence is promising but remains mainly cellular and rodent-based.

F1 sperm endpoints should be interpreted as intergenerational outcomes when exposure occurs during gestation.

Sperm count, motility, morphology and viability require companion hormone, histology and molecular endpoints.

Flavanone research should complement exposure reduction and chemical regulation, not replace them.

## Résumé

Le bisphénol A (BPA) a été associé, dans des études épidémiologiques, à des altérations des hormones de la reproduction masculine et des paramètres spermatiques; dans les modèles expérimentaux, il induit des atteintes endocriniennes, oxydatives, mitochondriales, apoptotiques et développementales. Cette revue narrative évalue séparément les données probantes propres au BPA concernant la naringénine et la naringine, ainsi que les données complémentaires issues d’autres modèles de stress reproductif. Elle distingue également les effets observés chez la génération F1 directement exposée de ceux relevant d’une transmission transgénérationnelle, et met en relation les paramètres spermatiques avec les données hormonales, histologiques et moléculaires. Cette revue narrative synthétise les données publiées principalement entre janvier 2019 et septembre 2026, tout en intégrant des études antérieures présentant un intérêt mécanistique direct. Les données ont été recensées au moyen de recherches ciblées dans PubMed, des sources indexées dans Scopus, Google Scholar et les bases de données des éditeurs, puis organisées selon la période d’exposition, le mécanisme biologique, le type de modèle et le paramètre reproductif étudié. Les données les plus probantes concernant la toxicité du BPA pour la reproduction masculine proviennent de résultats convergents issus d’études cellulaires et animales, étayés par des études observationnelles chez l’être humain, bien qu’hétérogènes. Les données les plus solides concernant la naringénine ou la naringine sont précliniques et comprennent des modèles de cellules de Sertoli et des modèles chez le rat spécifiquement exposés au BPA, ainsi que des modèles connexes de stress oxydatif, métabolique et induit par les phtalates. Dans l’ensemble de la littérature préclinique, ces flavanones modulent les paramètres redox, mitochondriaux, inflammatoires et stéroïdogènes. Toutefois, les différences de modèle, de dose, de voie d’administration et de biodisponibilité empêchent toute comparaison quantitative. Les données disponibles justifient la poursuite des recherches mécanistiques, mais non une supplémentation Clinique. La réduction de l’exposition demeure la principale approche de santé publique.

## Introduction

Male reproductive development spans fetal life, infancy, puberty and early adulthood, when Sertoli-cell number, Leydig-cell function, germ-cell survival, meiosis, the blood-testis barrier and hypothalamic-pituitary-gonadal signalling are established. Experimental and clinical reviews show that environmental exposures during these windows can alter later reproductive outcomes, although effects depend on dose, timing, route and biological stage [9, 18, 31]. Meiosis itself depends on regulated protein turnover and protein kinase A stability, as illustrated by RNF216 studies in testes; this mechanistic evidence is not evidence of a BPA effect [24].

BPA is widely used in polycarbonate plastics, epoxy resins, thermal papers and food-contact materials. Regulatory interpretations differ: the European Food Safety Authority lowered the tolerable daily intake in 2023, whereas the United States Food and Drug Administration states that approved food-contact uses are safe at current exposure levels [14, 49]. This review therefore separates evidence of biological hazard from exposure realism and human causal inference.

Experimental studies link BPA to endocrine signalling, steroidogenesis, oxidative stress, mitochondrial dysfunction and cell-death pathways [2, 10, 12]. Human findings remain inconsistent. A meta-analysis of nine studies (2,399 men) found a small inverse association with motility that was not robust after adjustment for publication bias and sensitivity analysis [11], whereas a later synthesis of 18 studies reported small inverse associations with sperm concentration and total count but no robust association with below-reference semen quality [27].

Developmental studies add an offspring perspective. In one mouse experiment, maternal gavage with 50 mg/kg/day BPA from embryonic day 0.5 to 18.5 was followed by lower testis weight, testosterone, sperm count, motility, and acrosomal integrity in F1 males, together with impaired sperm-oocyte binding and early embryo development [53]. Because F1 animals were directly exposed in utero, these are intergenerational findings and do not by themselves demonstrate transgenerational inheritance.

Naringenin is a citrus flavanone, and naringin is a glycoside metabolized partly to naringenin. BPA-specific evidence is sparse: naringenin reduced oxidative and mitochondrial injury in BPA-exposed mouse Sertoli cells, and naringin improved several testicular outcomes in an adult-rat BPA model [4, 15, 22]. Findings from phthalate, metabolic, cancer, and other toxicological models may identify shared pathways but cannot establish protection against BPA [5, 33, 54].

Previous reviews have examined BPA and male fertility, epidemiology, antioxidant interventions or testicular immune disruption [3, 10, 28, 51]. The present review is narrower and study-level: it jointly appraises naringenin and naringin in BPA models, labels indirect non-BPA evidence, distinguishes intergenerational from transgenerational claims, and maps sperm outcomes to endocrine, histological, and molecular measures. This scope avoids duplicating broad reviews of BPA mechanisms or antioxidants and tests whether these two flavanones have direct BPA-specific support.

## Narrative review methodology

This narrative review integrates mechanistic, experimental and selected epidemiological evidence on BPA-related male reproductive toxicity and flavanone intervention studies. It emphasizes the directness of each model and the interpretation of preclinical and developmental endpoints.

Literature was identified through targeted searches of PubMed, Google Scholar, Scopus-indexed journal pages and publisher databases. Searches covered January 2019 through September 2026; older studies were retained when they provided direct mechanistic, model-specific or foundational value. Search terms, used alone and in combination, included bisphenol A, BPA, male fertility, sperm quality, sperm count, sperm motility, testosterone, Leydig cell, Sertoli cell, oxidative stress, mitochondrial dysfunction, apoptosis, offspring, prenatal exposure, endocrine-disrupting chemicals, naringenin, naringin, flavonoids, testicular toxicity, reproductive toxicity and phytochemicals. The eight additional references supplied during revision were screened under the same relevance criteria and cited with explicit qualification when their models were indirect.

Studies were eligible for core synthesis when they addressed BPA or bisphenol analogues and male reproductive outcomes, mechanisms relevant to sperm production or function, naringenin or naringin in male reproductive stress models, or developmental male-reproductive exposure designs. Studies restricted to female fertility, uterine immunology, unrelated cancers or non-reproductive outcomes were not treated as evidence of male BPA toxicity or flavanone efficacy; where cited, they define biological context or the limits of cross-model inference [16, 19, 32, 52]. Reference lists of key reviews were screened for mechanistically important studies (Table 1).

Because the search was narrative, no PRISMA flow diagram, formal risk-of-bias tool or pooled quantitative analysis was used. To improve transparency, the evidence was interpreted according to four criteria: relevance to male reproductive biology, directness of the BPA or flavanone exposure model, quality of endpoint integration and translational plausibility (see Table 2). Human epidemiological findings were treated as important for public-health relevance but not as proof of causality. Cell and animal studies were treated as valuable for mechanism but not as evidence of clinical efficacy. F1 offspring data were interpreted as intergenerational evidence when exposure occurred during gestation, whereas true transgenerational claims were reserved for designs extending to F2 or F3 generations, depending on exposure timing.

**Table 1:** Narrative search and evidence-selection framework

Element	Specification used in this review
Databases and sources	PubMed, Google Scholar, Scopus-indexed journal pages and publisher databases.
Date range	January 2019 to September 2026, with older studies retained when directly relevant.
Core concepts	BPA/bisphenol analogues; male reproductive outcomes; naringenin/naringin; oxidative, mitochondrial, endocrine and developmental mechanisms.
Included evidence	Core synthesis: male BPA/bisphenol studies and male reproductive flavanone models. Context-only references were explicitly identified as indirect.
Excluded evidence	Excluded from efficacy inference: female-only, uterine, unrelated cancer and non-reproductive evidence; general toxicology without interpretable male reproductive endpoints.
Interpretive approach	Narrative synthesis; directness of model, endpoint integration, exposure realism and translational limits considered. No formal risk-of-bias scoring or meta-analysis.

## Overview of BPA as a male reproductive toxicant

BPA is a synthetic compound used in the production of polycarbonate plastics and epoxy resins. Its relevance to reproductive health arises from widespread exposure, hormonal activity and the ability to influence multiple cellular stress pathways [10]. In male reproductive biology, BPA is not limited to one site of action. It can interact with estrogen receptors, androgen-related signalling, steroidogenic pathways, Sertoli cell function, Leydig cell activity, germ-cell survival, blood-testis barrier integrity, sperm maturation and sperm molecular composition [12, 34].

One difficulty in interpreting BPA evidence is that endocrine-disrupting chemicals can show stage-dependent effects, mixture interactions and non-monotonic dose-response patterns. High-dose animal studies are useful for mechanisms but do not always reflect ordinary background exposure. Low-dose epidemiological studies are closer to real-world exposure but are affected by short biological half-life, single-spot urinary measures, co-exposures, fertility selection, and confounding. These limitations explain why mechanistic evidence is strong while human semen results remain variable across studies.

Human and experimental evidence converges on several domains but not on a single causal estimate. The two available meta-analyses differ in included studies and conclusions: Castellini et al. [11] judged the pooled association with semen quality clinically negligible or uncertain after sensitivity analyses, while Lü et al. [27] reported inverse pooled associations with sperm concentration (β = -0.03, 95% CI -0.06 to -0.01) and total count (β = -0.05, 95% CI -0.08 to -0.02). This divergence supports cautious interpretation rather than a uniform human effect claim.

Recent human studies also show endpoint-specific results. In 195 Spanish men, higher urinary BPA and BPF were associated with lower sperm vitality [29]. In 158 healthy Spanish university students, BPA was not significantly associated with sperm DNA fragmentation in a chemical-mixture analysis [20]. Conversely, repeated urinary measurements in 474 Chinese infertility-clinic patients linked BPA with greater comet-assay tail length and tail distributed moment (p for trend < 0.05) [25]. These cross-sectional findings differ in population, exposure assessment and outcome and cannot establish causality.

## Mechanisms linking BPA exposure to male reproductive dysfunction

### Endocrine disruption and steroidogenic impairment

**Fig. 1:**
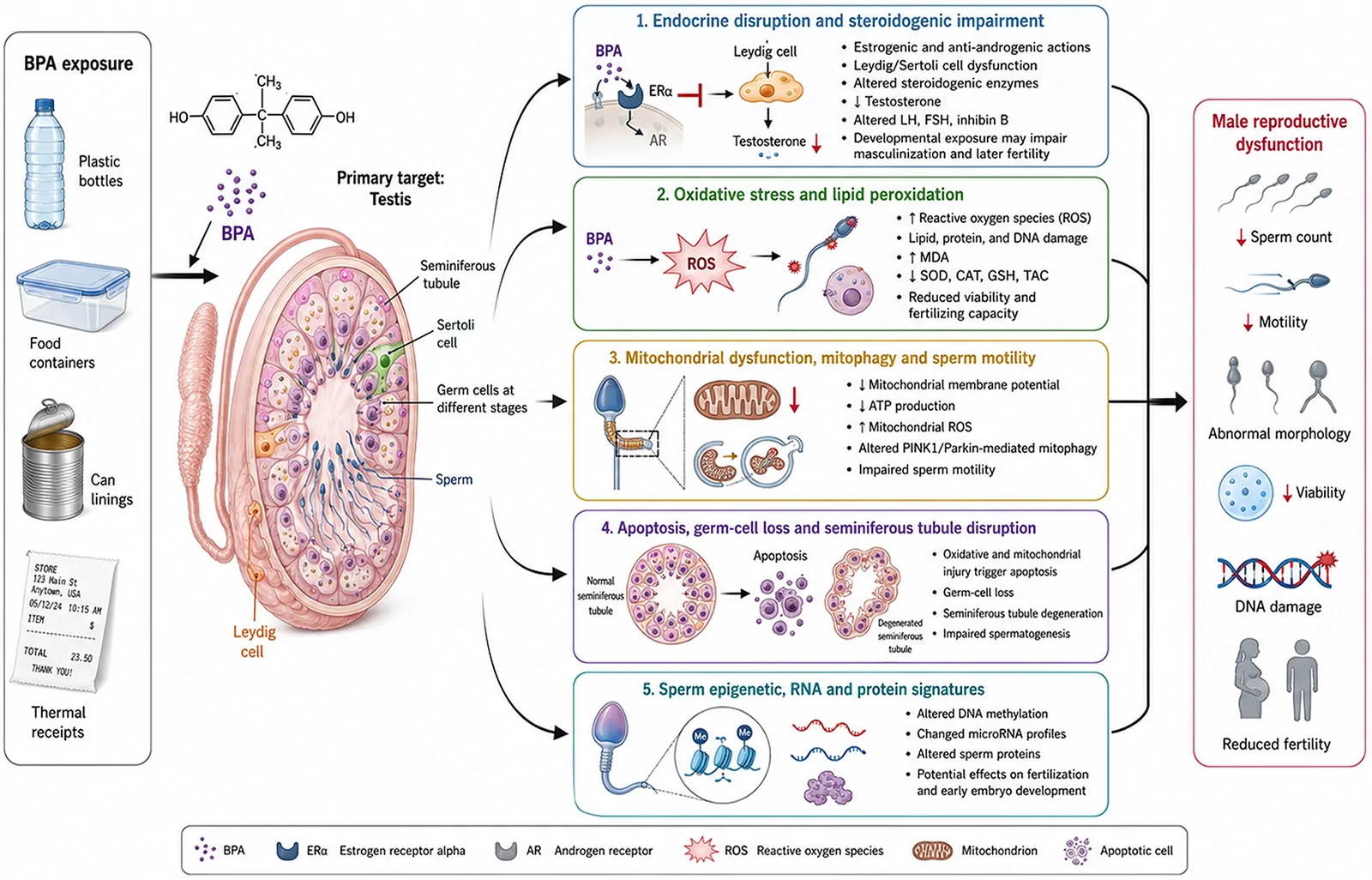
Mechanisms linking BPA exposure to male reproductive dysfunction

Testosterone is essential for spermatogenesis, epididymal maturation, libido and normal male reproductive function. Leydig cells synthesize testosterone under luteinising hormone support, while Sertoli cells respond to endocrine signals to support germ-cell development, as shown in Fig. 1. BPA disrupts this system in experimental models through estrogenic and anti-androgenic actions, altered steroidogenic enzyme expression and interference with Leydig cell maturation. Experimental studies report reduced testosterone concentrations and altered genes or proteins involved in steroid hormone synthesis after BPA exposure [12, 37, 53].

Endocrine disruption may be especially consequential during development, but the evidence varies by model and dose. Developmental BPA experiments have reported lower testosterone, reduced testis weight and impaired spermatogenesis or sperm function in offspring. Independent work showing that RNF216 regulates meiotic progression and protein kinase A stability in testes helps identify plausible endpoints, but it did not test BPA [24]. Hormone profiles should therefore accompany histology and sperm measurements in BPA studies.

### Oxidative stress and lipid peroxidation

Oxidative stress is one of the most consistent pathways connecting BPA exposure to testicular and sperm injury. Sperm cells are particularly vulnerable because their plasma membranes contain polyunsaturated fatty acids and because mature sperm have limited cytoplasmic antioxidant reserve. Excess reactive oxygen species can damage lipids, proteins, mitochondrial membranes and DNA, leading to reduced motility, abnormal morphology, decreased viability and impaired fertilising capacity.

In testicular tissue, oxidative stress can affect Sertoli-cell support, Leydig-cell steroidogenesis and germ-cell survival. Malondialdehyde, superoxide dismutase, catalase, glutathione and total antioxidant capacity are useful companion endpoints in BPA studies [30, 39]. Evidence that glutamate and aspartate supported antioxidant and immune defences in boar testes and epididymides further illustrates the dependence of male reproductive tissue on redox homeostasis, although that nutritional model did not involve BPA or flavanones [46].

### Mitochondrial dysfunction, mitophagy and sperm motility

Mitochondria are central to sperm motility, germ-cell development and steroidogenic function. BPA-induced mitochondrial dysfunction has been linked with impaired membrane potential, reduced ATP production, increased mitochondrial reactive oxygen species and altered mitophagy [41]. These changes are relevant to sperm motility because progressive movement depends on coordinated energy production, flagellar function and membrane integrity.

Ryu et al., [37] reported that BPA disturbed hormonal levels and testis mitochondrial activity in association with reduced male fertility. At the cell level, naringenin studies in BPA-exposed Sertoli cells identify mitochondrial membrane potential and Pink1/Parkin-mediated mitophagy as relevant endpoints [22]. These findings support inclusion of mitochondrial measures in studies of BPA-related sperm dysfunction.

### Apoptosis, germ-cell loss and seminiferous tubule disruption

Normal spermatogenesis requires a balance between germ-cell proliferation, differentiation and controlled apoptosis. BPA can shift this balance toward germ-cell loss by inducing oxidative stress, mitochondrial injury, inflammatory signalling and apoptotic pathways. Excess apoptosis in spermatogonia, spermatocytes or spermatids reduces spermatogenic output and alters seminiferous tubule architecture.

Histological evidence is important because sperm variables alone cannot identify the site of injury. Reduced sperm count can reflect Sertoli cell dysfunction, germ-cell apoptosis, endocrine insufficiency, tubular degeneration or epididymal maturation defects. Narrative synthesis of BPA studies therefore requires integration of semen endpoints with testicular histology, hormone profiles and molecular markers.

### Sperm epigenetic, RNA and protein signatures

Conventional semen variables do not capture all molecular changes in sperm. Epigenetic marks, non-coding RNAs and proteins participate in chromatin organization, fertilization and early development [26, 44, 47]. These endpoints may be useful in reproductive toxicology, but an altered molecular profile should not be assumed to predict infertility without functional validation.

In a cross-sectional cohort of 102 Portuguese men, seminal-plasma BPA was not significantly correlated with conventional semen parameters; small-RNA and proteomic subsets nevertheless showed differential molecular profiles [40]. Together with the contrasting sperm-DNA findings of Kiwitt-Cárdenas et al. [20] and Li et al. [25], these data suggest that molecular endpoints may be exposure-sensitive but are not yet validated surrogates for infertility or embryo outcomes.

Accordingly, sperm epigenetic, transcriptomic, and proteomic measures should complement rather than replace standardized semen analysis, hormones, histology, and functional fertility outcomes [6, 43]. Persistence across generations requires appropriately designed F2 or F3 studies; molecular changes in F1 sperm alone do not prove transgenerational inheritance.

As illustrated in Fig. 1, BPA exposure from plastic bottles, food containers, can linings and thermal receipts targets the testis and disrupts male reproductive function through interconnected endocrine, oxidative, mitochondrial, apoptotic and molecular pathways. BPA impairs Leydig and Sertoli cell function, reduces testosterone synthesis, increases reactive oxygen species and lipid peroxidation, damages mitochondrial activity and mitophagy, promotes germ-cell apoptosis and seminiferous tubule degeneration, and alters sperm epigenetic, RNA and protein signatures. These mechanisms collectively contribute to reduced sperm count, impaired motility, abnormal morphology, decreased viability, DNA damage and reduced fertility.

## Developmental exposure and offspring reproductive programming

Fetal and early postnatal development includes germ-cell differentiation, Sertoli-cell proliferation, Leydig-cell maturation and establishment of hypothalamic-pituitary-gonadal signalling [7, 18]. Disruption during these windows may alter adult reproductive function, but effects depend on exposure timing, dose and model. F1 outcomes after gestational exposure are intergenerational because the fetus was directly exposed [9].

Evidence from individual prenatal or perinatal BPA experiments reports adverse male-reproductive outcomes, but it is not uniform enough to claim that every exposure produces persistent dysfunction. Zhang et al. [53] reported statistically significant reductions in testis weight, serum testosterone, sperm count, motility measures and acrosomal integrity in adult F1 mice after maternal exposure to 50 mg/kg/day BPA, with reduced sperm-oocyte binding and two-cell and eight-cell embryo development (*p* < 0.05). The high experimental dose limits extrapolation to typical human exposure.

Developmental BPA studies also report epigenetic, oxidative, mitochondrial, blood-testis-barrier and apoptotic changes [8, 17, 50, 38, 55]. These findings support mechanistic hypotheses, not a conclusion that BPA permanently programs reproductive health in all exposed offspring. Replication at environmentally relevant internal doses and follow-up beyond F1 are needed.

## Naringenin and naringin: biological profile and reproductive relevance

### Chemical, nutritional and pharmacokinetic context

Naringenin is abundant in citrus fruits, while naringin is a glycosylated flavanone converted partly to naringenin by intestinal microflora [45]. In a randomized single-dose crossover trial of 18 healthy adults, 150–900 mg naringenin produced dose-related systemic exposure without relevant short-term adverse events; the reported half-life was approximately 2.7–3.0 h [36]. This study informs exposure and short-term safety, not male fertility efficacy. Cervical cancer pathway experiments remain tissue-specific and are not reproductive evidence [54].

For male reproductive toxicology, the relevant question is whether administered flavanone doses produce sustained, unconjugated concentrations in testicular tissue that modify injury without adverse effects. The human pharmacokinetic trial did not measure reproductive outcomes or testicular exposure [36]. BPA-specific studies address Sertoli-cell oxidative stress and mitophagy or rat testicular outcomes, whereas evidence from other models remains indirect.

### Evidence from BPA-specific models

Khorsandi et al. [22] exposed mouse TM4 Sertoli cells to 0.8 µM BPA for 24 h and tested naringenin at 10, 20 and 50 µg/mL. BPA reduced viability (*p* = 0.004), mitochondrial membrane potential (*p* < 0.001) and antioxidant biomarkers (*p* = 0.003), and increased reactive oxygen species, malondialdehyde, PINK1 and Parkin (*p* < 0.001). Naringenin at 20 and 50 µg/mL improved viability and membrane potential (*p* = 0.034), and concentration-dependent treatment reduced PINK1/Parkin expression (*p* = 0.024). This is direct BPA-specific mechanistic evidence in one immortalized cell line, not evidence of restored fertility.

Alboghobeish et al. [4] assigned 42 adult male Wistar rats to six groups (*n* = 7/group) and administered BPA 50 mg/kg with or without oral naringin 40, 80 or 160 mg/kg for 30 days. Naringin improved several BPA-altered biochemical, hormonal, sperm-count and histological measures (reported as statistically significant in the article). The single study, high BPA dose and absence of replication in humans limit the strength of any efficacy claim.

### Evidence from other reproductive stress models

Evidence from non-BPA models is supportive only. In 42 adult male Wistar rats (*n* = 7/group), oral naringin 80 mg/kg given three times weekly for eight weeks improved testosterone, sperm count, motility, viability, redox markers and histology after di-n-butyl phthalate exposure (250 or 500 mg/kg; *p* < 0.05) [5]. In 30 adult male Wistar rats (*n* = 6/group), naringin 50 mg/kg for 28 days improved selected hormonal, testicular-injury, redox, and histological measures in a high-fat-diet/streptozotocin diabetes model (*p* < 0.05) [33]. Neither study tested BPA.

Other reproductive-toxicology work, including reviews of benzo[a]pyrene, shows that oxidative, endocrine, and germ-cell pathways recur across toxicants [50]. Shared pathways provide biological context but do not demonstrate a BPA-specific chemical interaction or justify pooling effect sizes across toxicants.

## Functional sperm endpoints for future BPA and flavanone studies

### Sperm count

Sperm count is a population-level output of spermatogenesis [42]. It depends on Sertoli cell support, germ-cell proliferation, meiosis, spermiogenesis, endocrine signalling and seminiferous tubular integrity. BPA-associated reduction in sperm count reflects impaired testicular production capacity in many experimental models [1, 23]. In developmental exposure studies, reduced F1 sperm count is interpreted as an adult reproductive outcome of earlier testicular injury [35, 48].

Preservation of sperm count in naringenin or naringin intervention studies indicates protection of spermatogenic output in that model [13, 21]. Count alone is insufficient. A high sperm count with poor motility or high abnormal morphology does not necessarily translate into fertility competence. Therefore, sperm count should be interpreted alongside motility, morphology, viability, hormone concentrations and histology.

### Sperm motility

Sperm motility is one of the most functionally informative semen endpoints because it reflects energy production, mitochondrial health, axonemal structure, membrane fluidity and oxidative status. BPA reduces motility in experimental models through mitochondrial damage, lipid peroxidation and altered sperm protein profiles. Naringenin-mediated preservation of mitochondrial membrane potential in Sertoli cells provides a mechanistic rationale for including motility in flavanone intervention studies [22].

Future studies should distinguish total motility from progressive motility and should include computer-assisted sperm analysis when possible. Motility changes are highly sensitive to laboratory handling, species differences and timing after collection. Standardization is therefore essential for comparing BPA and phytochemical studies.

### Sperm morphology and viability

Sperm morphology reflects the quality of germ-cell differentiation, nuclear shaping, acrosome formation, flagellar assembly and cytoplasmic remodeling. BPA-associated abnormal morphology arises from oxidative damage, disrupted spermiogenesis, defects in meiosis, chromatin injury, and Sertoli cell dysfunction in experimental settings. Zhang et al. [53] linked prenatal BPA exposure with meiotic disruption and sperm functional defects in offspring, reinforcing the relevance of morphology as a developmental endpoint.

Sperm viability reflects membrane integrity and resistance to cellular damage. Oxidative stress compromises membranes through lipid peroxidation, while mitochondrial dysfunction and apoptosis-related pathways reduce sperm survival. Viability is therefore useful for identifying whether an intervention stabilizes sperm membranes and reduces oxidative cell injury. Because viability can improve even when spermatogenic output remains compromised, it should not be used as the sole indicator of fertility recovery.

## Integrated evidence summary and interpretive framework

**Fig. 2:**
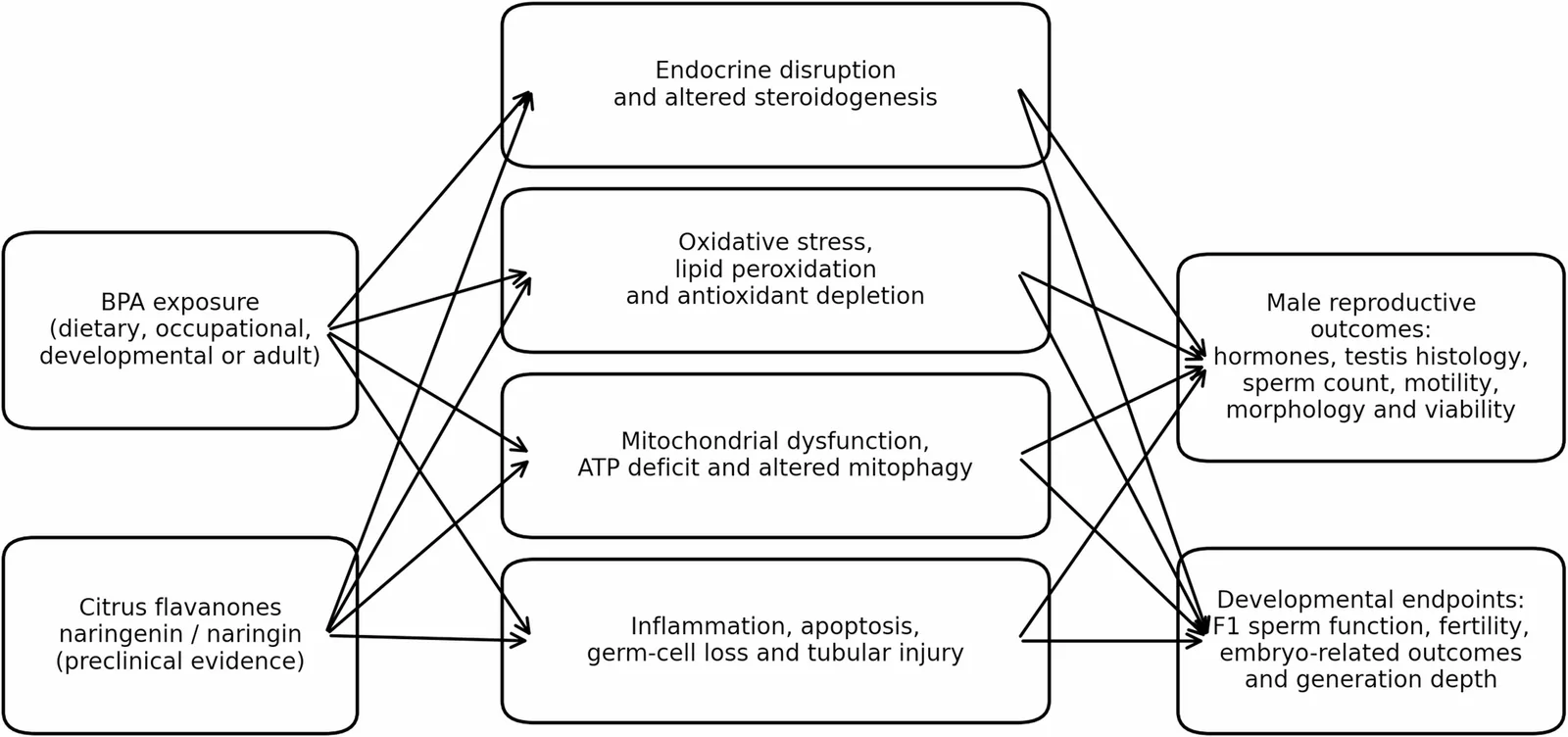
Mechanistic framework for BPA-induced male reproductive toxicity and reported flavanone responses. BPA exposure is associated with endocrine disruption, oxidative stress, mitochondrial dysfunction, apoptosis, germ-cell loss and altered sperm function. Naringenin and naringin studies report effects on redox balance, mitochondrial activity, inflammatory signalling, apoptosis and steroidogenic pathways in selected cell and animal models

The evidence forms two related but unequal streams as presented in Fig. 2. BPA studies describe human associations and experimental endocrine, redox, mitochondrial, germ-cell and developmental outcomes. Flavanone studies report changes in some of the same pathways, but direct BPA-specific evidence remains limited to one Sertoli-cell naringenin study and one rat naringin study. Table 2 presents all representative studies in one table and labels each row by evidence category so that indirect non-BPA models are not interpreted as direct protection against BPA.

A narrative synthesis is useful because BPA studies and phytochemical studies are diverse in model, dose, route, timing and endpoint selection. Direct comparison across studies is difficult, but evidence can be organized around recurring biological domains: steroidogenesis, oxidative stress, mitochondrial integrity, apoptosis, testicular histology and sperm function. This approach avoids broad treatment claims and keeps the review grounded in measured outcomes.

Sperm count reflects spermatogenic output and Sertoli-Leydig-germ cell coordination. Motility reflects mitochondrial competence, ATP support, flagellar function and membrane fluidity. Morphology reflects spermiogenesis, chromatin packaging and germ-cell differentiation. Viability reflects membrane integrity and cellular survival. When these outcomes are measured together with hormones and histology, they provide a practical picture of reproductive injury and recovery in experimental studies.

**Table 2:** Representative human, BPA-specific and indirect flavanone evidence informing the narrative synthesis

Evidence category and study	Model and design	Exposure or intervention	Key reproductive finding	Interpretive value
Direct BPA-naringin animal | Alboghobeish et al. [4]	Adult male Wistar rats; six groups; *n* = 42 (7/group).	BPA 50 mg/kg/day; oral naringin 40, 80 or 160 mg/kg/day; 30 days; naringin-only 160 mg/kg group.	Naringin significantly improved BPA-altered epididymal sperm count, hormones, antioxidant enzymes, lipid peroxidation and testicular histology; exact effect sizes were not consistently reported in the abstract.	Direct BPA-naringin animal evidence; high BPA dose and single study limit translation.
Direct BPA animal | Ryu et al. [37]	Male CD-1 (ICR) mice, 4 weeks old; control, 5 and 50 mg/kg groups; *n* = 20/group. Main mechanistic comparisons used control vs. 50 mg/kg.	BPA in corn oil by oral gavage daily from age 6 to 11 weeks (6 weeks); 1-week stabilization before sampling.	At 50 mg/kg, FSH increased and LH/testosterone decreased; testis lesions, mitochondrial-protein changes, and lower embryo cleavage/blastocyst formation occurred (*p* < 0.05). Testis weight did not differ.	Direct BPA study; authors noted dose exceeded typical human exposure.
Direct BPA-naringenin cell | Khorsandi et al. [22]	Mouse TM4 Sertoli-cell laboratory study; independent experimental replicates reported, no animals.	BPA 0.8 µM for 24 h; naringenin 10, 20–50 µg/mL.	BPA reduced viability (*p* = 0.004), MMP (*p* < 0.001) and antioxidants (*p* = 0.003). Naringenin 20/50 µg/mL improved viability and MMP (*p* = 0.034) and reduced PINK1/Parkin expression (*p* = 0.024).	Direct BPA-naringenin mechanism; immortalized cell line and no fertility endpoint.
Human meta-analysis [27]	Systematic review/meta-analysis of 18 epidemiological studies (2010–2023); mostly cross-sectional/cohort populations; age and sample size varied.	Measured BPA, predominantly urinary; observational exposure, no intervention.	Inverse pooled associations with sperm concentration (β = -0.03, 95% CI -0.06 to -0.01; *p* = 0.003) and total count (β = -0.05, 95% CI -0.08 to -0.02; *p* < 0.001); below-reference semen-quality result was not robust.	Human synthesis; association does not establish causality.
Human observational [29]	Led-Fertyl cross-sectional study; 195 adult Spanish men.	Single urinary BPA, BPF and BPS measurements, creatinine-adjusted; no intervention.	Higher BPA and BPF were associated with lower sperm vitality. Highest vs. lowest quartile: BPA β = -6.90% (95% CI -11.60 to -2.15) and BPF β = -9.68% (-14.43 to -4.94).	Human analogue evidence; single-spot urine and cross-sectional design limit causality.
Human observational [40]	Cross-sectional cohort of 102 Portuguese men; small-RNA subset *n* = 15; proteomics subset *n* = 20.	Seminal plasma BPA measured by LC-MS/MS; observational gradients; no administered dose or duration.	No significant correlation with conventional semen parameters; differential sperm microRNAs and proteins were identified across BPA levels (study thresholds *p* < 0.05).	Candidate molecular biomarkers; small subsets and cross-sectional design.
Direct prenatal BPA animal [53]	Pregnant ICR mice, control *n* = 15 and BPA *n* = 15; adult F1 males assessed mainly at PND70.	Maternal BPA 50 mg/kg/day by oral gavage, embryonic day 0.5–18.5; corn-oil control.	F1 testis weight, testosterone, sperm count, motility, and acrosomal integrity decreased; sperm-oocyte binding and 2-/8-cell embryo development decreased (*p* < 0.05).	Direct prenatal BPA evidence; intergenerational F1 result at a high experimental dose.
Human meta-analysis [11]	Meta-analysis of 9 observational studies; 2,399 men.	Urinary BPA; multivariable regression estimates for standard semen measures.	Motility: pooled β = -0.82 (95% CI -1.51 to -0.12; *p* = 0.02), but significance was lost after publication-bias adjustment and influential-study sensitivity analyses.	Balanced human synthesis; overall association judged weak or clinically negligible.
Human observational [20]	Cross-sectional mixture study; 158 healthy male university students in Spain.	Same-day spot urine and semen; BPA, parabens and benzophenones; Bayesian kernel machine regression.	No significant association between BPA and sperm DNA-fragmentation index.	Null BPA result; single urine sample and mixture design limit inference.
Human observational [25]	Cross-sectional study; 474 men recruited from a Chinese infertility clinic.	Repeated urinary BPA, BPF and BPS measurements; sperm DNA damage assessed by comet assay.	Higher BPA was associated with greater tail length and tail distribution moment (both p for trend < 0.05).	Repeated exposure measures strengthen assessment, but clinic selection and cross-sectional design limit causality.
Indirect non-BPA model [5]	Adult male Wistar rats, 3 months old; *n* = 42 (7/group).	Oral naringin 80 mg/kg, 1 h before DBP 250 or 500 mg/kg; gavage 3 times/week for 8 weeks.	Naringin improved testosterone, sperm count, motility, viability, redox markers and seminiferous histology versus DBP groups (*p* < 0.05).	Indirect: phthalate, not BPA.
Indirect non-BPA model [33]	Adult male Wistar rats; *n* = 30 (6/group); HFD/streptozotocin diabetes model.	Oral naringin 50 mg/kg/day for 28 days after diabetes induction; comparator groups included orange-peel extract and metformin.	Selected reproductive hormones, testicular injury/redox markers and histology improved versus untreated diabetes (*p* < 0.05); several effects were smaller than comparators.	Indirect: metabolic disease, not BPA.

## Translational interpretation and public-health relevance

Translation to humans is limited by mixtures, diet, repeated low-level exposure, metabolism and the gap between administered dose and active testicular concentration. A small single-dose trial supports short-term systemic exposure and tolerability of naringenin in healthy adults, but it did not assess semen, hormones, fertility, or long-term safety [36]. Current evidence therefore does not establish a safe or effective reproductive dose or prevention of BPA-related infertility.

The defensible interpretation is mechanistic: the studies identify redox, mitochondrial, endocrine and histological endpoints for further testing. They do not support prescribing naringenin or naringin for fertility. Exposure prevention and evaluation of replacement bisphenols remain the primary public-health priorities.

Public-health conclusions should also remain distinct from evidence in other reproductive contexts. Studies of female ART outcomes after Omicron infection, uterine T-cell phenotypes, inactivated COVID-19 vaccination in couples undergoing ART, and TRIM21-dependent oocyte metabolism address important reproductive questions but do not provide evidence about male BPA toxicity or flavanone protection [16, 19, 32, 52].

## Research gaps and future directions

Several gaps limit the field. First, dose realism is insufficient. Many animal studies use BPA doses that are mechanistically useful but higher than typical human exposure. Future designs should include environmentally relevant doses, higher mechanistic doses, and internal exposure measurements where possible. Second, timing requires clearer attention. Gestational, lactational, pubertal, and adult exposures should not be pooled without acknowledging biological differences.

Third, generation depth must improve. F1 offspring studies are necessary for developmental programming but are not sufficient for strong transgenerational claims. F2 and F3 designs are needed when authors wish to discuss inheritance beyond directly exposed generations. Fourth, endpoint integration is often incomplete. Sperm variables should be measured with hormones, histology, oxidative stress, mitochondrial function, apoptosis and molecular sperm cargo.

Fifth, phytochemical comparison is needed to determine whether reported effects are flavanone-specific or shared across antioxidant classes. Sixth, pharmacokinetic bridging should measure parent compounds, conjugated metabolites and testicular concentrations after repeated oral dosing. Existing human single-dose data establish systemic exposure but not reproductive target engagement [36].

Seventh, reproductive outcomes should extend beyond semen analysis. Fertilisation rate, sperm-egg binding, acrosome reaction, embryo development, pregnancy rate and offspring health can provide stronger evidence of functional fertility protection. Finally, human studies should avoid supplement claims until carefully designed clinical or observational research has established safety, feasibility and relevance.

## Experimental considerations for future studies

Future experimental studies should include at least four groups: control, BPA only, naringenin or naringin only, and BPA plus naringenin or naringin. If developmental exposure is the focus, exposure should be timed to gestation and/or lactation, with male F1 offspring assessed at reproductive maturity. If post-exposure recovery is the focus, the phytochemical should be administered after BPA exposure has ended, with comparison of pubertal and adult intervention windows.

Core outcomes should include testis weight, epididymal sperm count, total and progressive motility, morphology, viability, testosterone, luteinising hormone, follicle-stimulating hormone, inhibin B, testicular histology, seminiferous tubule diameter, oxidative stress markers, mitochondrial membrane potential, apoptosis markers, mitophagy markers and sperm DNA fragmentation. Advanced endpoints can include sperm proteomics, small RNA profiling, acrosome integrity, sperm-egg binding and early embryo development.

Results should be interpreted within the measured domain. Improvement in motility or viability does not establish restoration of spermatogenesis, and normalization relative to a high-dose toxicant group does not establish clinical benefit. Functional fertility, adverse effects, exposure biomarkers and pharmacokinetics should be reported alongside statistical significance and effect sizes.

## Limitations of this narrative review

This review has limitations inherent to narrative synthesis. The literature search was targeted rather than systematic, and no pooled effect sizes were calculated. Study selection emphasized scientific relevance, mechanistic value, and recency rather than exhaustive inclusion. The evidence tables are representative and not complete evidence maps. Differences in species, BPA dose, route of exposure, timing, duration, naringenin or naringin dose, and semen-analysis methods limit direct comparison across studies.

The intervention evidence is especially limited. Only a few immortalized mouse Sertoli-cell studies of naringenin and one adult-rat study of naringin directly used BPA. Neither established a human fertility benefit, and the rat BPA dose exceeded typical exposure. The available naringenin clinical trial was small, single-dose, and not reproductive; it cannot establish repeated-dose safety, testicular exposure, naringin equivalence, or efficacy [36]. Human BPA studies remain vulnerable to exposure misclassification, residual confounding, reverse causation, and selection, even when repeated urine samples improve exposure assessment. These limitations preclude causal, therapeutic or dose recommendations. Therefore, the interpretive structure used in this review is intended to organize existing evidence and guide future endpoint selection, not to establish a therapeutic recommendation.

## Conclusion

BPA is associated with altered reproductive hormones and sperm-count measures in epidemiological synthesis and produces endocrine, oxidative, mitochondrial, histological and developmental injury in experimental models. However, human causal inference remains limited, and high-dose animal findings cannot be assumed to represent typical exposure. Naringenin and naringin modify relevant pathways in preclinical systems, but direct BPA-specific evidence comprises few Sertoli-cell experiments and few adult-rat study. Evidence from phthalate, metabolic and other toxicological models is indirect. These findings justify better-designed mechanistic studies with exposure-relevant doses, pharmacokinetics, integrated sperm and testicular endpoints, functional fertility outcomes and appropriate F2/F3 designs when transgenerational inheritance is claimed. They do not justify clinical supplementation; reducing bisphenol exposure remains the primary public-health approach.

## Data Availability

No datasets were generated or analysed during the current study.
